# Complete mitochondrial genome of a hen harrier *Circus cyaneus* (Accipitriformes: Accipitridae) from South Korea

**DOI:** 10.1080/23802359.2020.1860700

**Published:** 2021-01-19

**Authors:** Eun Hwa Choi, Gankhuyag Enkhtsetseg, Su Youn Baek, Jihye Hwang, Bia Park, Kuem Hee Jang, Shi Hyun Ryu, Ui Wook Hwang

**Affiliations:** a Department of Biology, Teachers College & Institute for Phylogenomics and Evolution, Kyungpook National University, Daegu, South Korea; b Institute for Korean Herb-Bio Convergence Promotion, Kyungpook National University, Daegu, South Korea; c Research Center for Endangered Species, National Institute of Ecology, Yeongyang, South Korea; d Strategic Planning Department, Nakdonggang National Institute of Biological Resources, Sangju, South Korea

**Keywords:** Complete mitochondrial genome, hen harrier, *Circus cyaneus*, Accipitridae, molecular phylogeny

## Abstract

A hen harrier *Circus cyaneus* (Accipitriformes: Accipitridae), a migrant raptor having a wide breeding range from Europe to Northeast Asia, migrates to more southerly areas (Southern Europe, China, Korea and Japan) in winter. In this study, the complete mitochondrial genome of *C. cyaneus* was completely sequenced and characterized. It was 20,173 bp in length being composed of 13 protein-coding genes, 22 transfer RNA genes, two ribosomal RNA genes and two control regions. It has a base composition of A (32.2%), G (12.6%), C (30.5%) and T (24.7%). The phylogenetic tree reconstructed based on the maximum likelihood (ML) method confirms that *C. cyaneus* places within the clade of the family Accipitridae in the monophyletic avian order Accipitriformes.

A hen harrier *Circus cyaneus* (Linnaeus, 1766) is a representative winter migratory bird and an endangered species by the Ministry of Environment, South Korea, belonging to the avian family Accipitridae (Order Accipitriformes), which has a wide range of habitats from Europe and North Asia to Russian Far East. In winter season, it migrates from Europe and Northwest Africa through Turkey and Middle East to Southeast China, Korean peninsula and Japan (Ferguson-Lees and Christie 2006).

The specimen of *C. cyaneus* used in the present study was collected from Dalseong wetland, Dalseong-gun, Daegu city, South Korea (35°49'02.4"N 128°29'26.9"E). The specimen was deposited under the voucher number GEIBGR0000289530 in the National Institute of Biological Resources (NIBR), South Korea. Total genomic DNA of *C. cyaneus* was extracted using QIAamp Tissue Kit (QIAGEN Co., Germany), and the mitochondrial genome was amplified by long range PCR method using Expand^TM^ Long Template PCR System (Roche Co., Germany). *12S rRNA* gene and four overlapping long fragments were amplified with specific primers shown in Table S1. Sequences were aligned and trimmed using the Clustal X2 program (Larkin et al. 2007) and BioEdit 7.0.9 program (Hall 1999). Protein-coding genes (PCGs), rRNAs, tRNAs and control region were characterized using NCBI Basic Local Alignment Search Tool (BLAST) and a program tRNAscan-SE (Chan and Lowe 2019).

The mitochondrial genome of *C. cyaneus* is 20,173 bp long (GenBank accession no. KU237286), which exhibits the same gene order with that of the order Accipitriformes. It contains total 37 genes including 13 PCGs (*COX1-3*, *ND1-6*, *ND4L*, *CYTB*, *ATP6* and *ATP8*), 2 rRNAs (*16S rRNA* and *12S rRNA*), 22 tRNAs and 2 control regions (CR and ψCR), of which 28 genes places on the heavy strand (H-strand) and the remaining 9 genes are located on the light strand (L-strand). The overall genome components and gene orders are identical to those of *C. cyaneus* published with the specimens of Inner Mongolia (Gao et al. 2018). The overall A + T content of *C. cyaneus* mitogenome is 56.9%: 53.7% for PCGs, 56.7% for tRNAs, 53.2% for rRNAs and 67.0% for CRs. In the PCGs (11,400bp long), 12 are encoded in the H-strand, and the only one (*CYTB*) is located in the L-strand. All PCGs use the conventional start codons ATN except for *COX1* using GTG which was also employed as the initiation-codon in other avian species (Slack et al. 2003). CR (3,571bp long) locates between *trnT* and *trnP*, which is divided into three domains of Domain I, Central Conserved Domain II, and Domain III. Domain II exhibits only five conserved boxes of F, E, D, C and b, and B-box, generally existing in Aves, is not found.

The maximum likelihood (ML) tree was reconstructed with 13 PCGs among 27 accipitriform species (Figure 1). The best fitting model mtVer + I+G4 was selected for the ML analysis. The ML tree confirms that *C. cyaneus* places within the clade of the family Accipitridae in the monophyletic avian order Accipitriformes. Within the family Accipitridae, there are found four different clades of Clade 1, 2, 3 and 4. In Clade 2, *C. cyaneus* from South Korea is grouped with *C*. *cyaneus* from Inner Mongolia within the monoclade of the genus *Circus*. *Accipiter* is a sister genus of *Circus*. The relationships of the four families of Accipitriformes are consistent with those of Burleigh et al. (2015) and Jiang et al. (2015).

**Figure 1.**
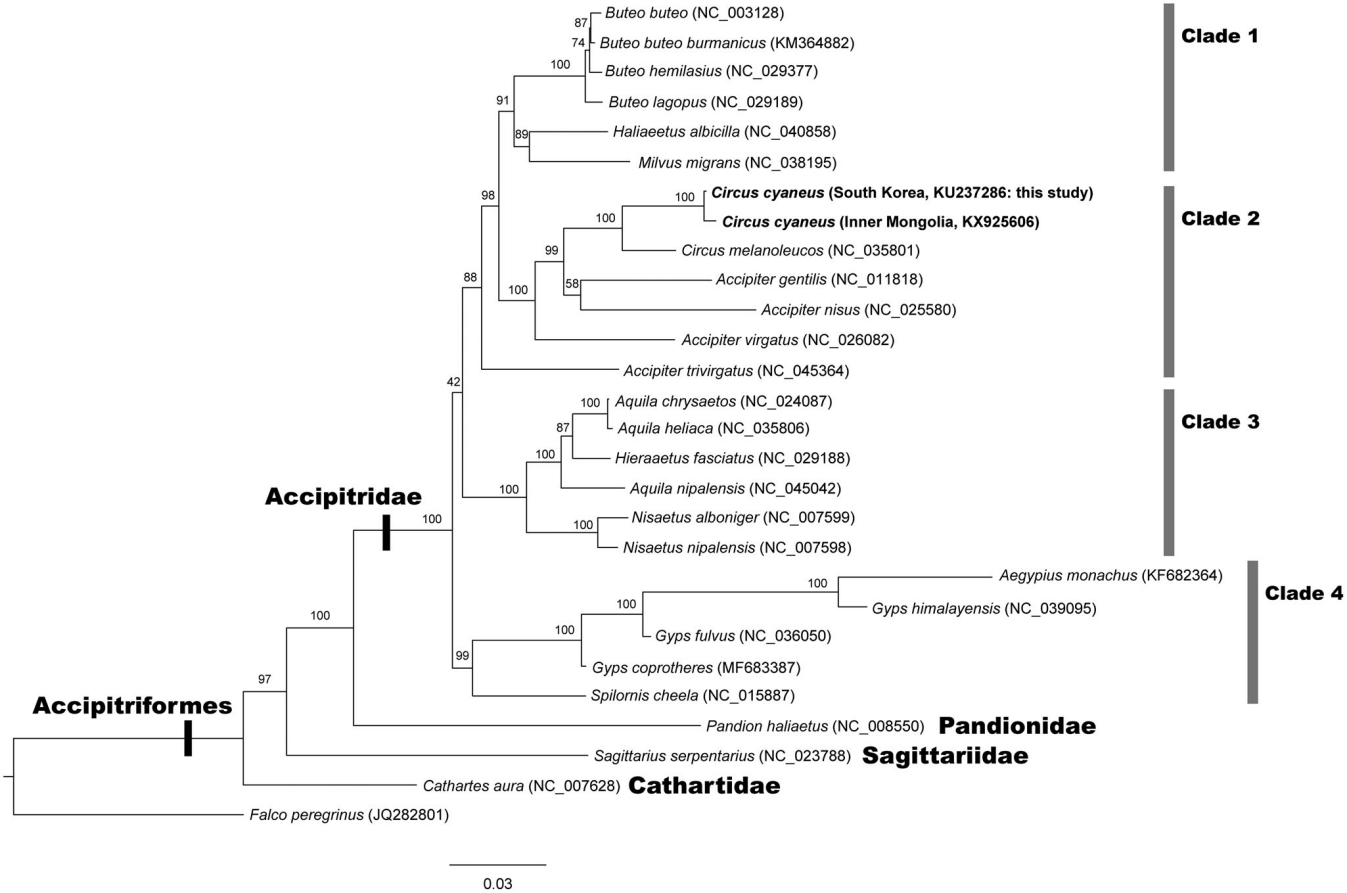
Maximum likelihood tree reconstructed with amino acid sequences of 13 mitochondrial protein-coding genes (PCGs) showing relationships among 27 accipitriform species. It indicates the monophylies of the family Accipitridae and the order Accipitriformes, and the existence of four different clades in the family Accipitridae. *C*. *cyaneus* (South Korea and Inner Mongolia) places within the Clade 2. *Falco peregrinus* (Order Falconiformes) was used as an outgroup. Branch supports are inferred from the ultrafast bootstrap method using IQ-TREE (Minh et al. 2020).

## Data Availability

The data that support the findings of this study are openly available in NCBI at https://www.ncbi.nlm.nih.gov/nuccore/KU237286.1. The information of the supplementary table was deposited in Figshae DB (https://doi.org/10.6084/m9.figshare.13139693.v1).
